# Mechanical and Conductive Properties of Cu Matrix Composites Reinforced by Oriented Carbon Nanotubes with Different Coatings

**DOI:** 10.3390/nano12020266

**Published:** 2022-01-14

**Authors:** Zhong Zheng, Anxin Yang, Jiafeng Tao, Jing Li, Wenqian Zhang, Xiuhong Li, Huan Xue

**Affiliations:** School of Mechanical Engineering, Hubei University of Technology, Wuhan 430068, China; anxin_0530@163.com (A.Y.); tjf18717191324@163.com (J.T.); lijing@hbut.edu.cn (J.L.); wenqian_zh@hbut.edu.cn (W.Z.); 20200005@hbut.edu.cn (X.L.); stonemechanics@163.com (H.X.)

**Keywords:** multi-walled carbon nanotubes, Cu matrix, interface, conductivity, ductility

## Abstract

Because of the dilemma that the current industrial Cu enhancement methods lead to a significant decline in conductivity and ductility, Cu matrix composites reinforced by oriented multi-walled carbon nanotubes (MWCNTs) were prepared through sintering, hot extrusion, and cold drawing. Before sintering, Ni, Cu, and Ni&Cu coatings were electroless plated on MWCNTs as the intermediate transition layer, and then they were mixed with Cu powder through a nitrogen bubbling assisted ultrasonic process. By analyzing the composition, microstructure, and formation mechanism of the interface between MWCNTs and the matrix, the influence and mechanism of the interface on the mechanical properties, conductivity, and ductility of the composites were explored. The results indicated that MWCNTs maintained a highly dispersed and highly consistent orientation in the Cu matrix. The coating on Ni@CNT was the densest, continuous, and complete. The Ni@CNTs/Cu composite had the greatest effect, while the Cu composite reinforced by MWCNT without coating had the smallest reduction in elongation and conductivity. The comprehensive performance of the Cu@CNTs/Cu composite was the most balanced, with an ultimate tensile strength that reached 373 MPa, while the ductility and conductivity were not excessively reduced. The axial electrical and thermal conductivity were 79.9 IACS % (International Annealed Copper Standard) and 376 W/mK, respectively.

## 1. Introduction

Copper has excellent electrical and thermal conductivity, good plastic formability, and is easy to manufacture, so it has become the best candidate material for electrical, thermoelectric, and electronic applications. However, with the rapid development of high-speed electric railways, lightweight automobile body manufacturing, new MEMS/NEMS (micro- or nanoelectromechanical system), and other fields in recent years, more stringent performance requirements have been put forward for key parts. These key parts include a high-speed railway electric contact line, resistance spot welding electrode for galvanized steel sheets, heat sink parts, and thermoelectric devices. Their conductive contact area is greatly decreased, the current density is greatly increased, and the heat emission condition deteriorates rapidly, which results in a significant decline in the stability of material parts. They are required to further enhance current-carrying or heat dissipation capacity with given sectional dimensions and structural strength or to reduce the sectional size to realize the miniaturization of components without sacrificing the intensity of texture or current-carrying and thermal conductivity. In other words, the strength, hardness, and electrical/thermal conductivity of the material need to be good, while the ductility and formability are acceptable. The following methods are commonly used to strengthen Cu in the current industry:

When Cu is strengthened by alloying (including solid solution, dispersion, and precipitation strengthening), its ductility or machinability will not be excessively reduced. However, the electron scattering caused by alloy elements will reduce its electrical/thermal conductivity.

The most mature method of reinforcing Cu is dispersion strengthening by reinforcements. The second phase mainly consists of ceramic reinforcing phases such as Al_2_O_3_ [1], TiB_2_ [2,3], and Zr_2_O_3_ and carbon reinforcing phases such as carbon fiber [4], graphite fiber [5], and graphite [6]. For example, the service life of Al_2_O_3_ dispersion strengthened Cu electrodes used in body-in-white welding, which were prepared by an internal oxidation method 4–10 times longer than that of ordinary chromium–zirconium Cu. However, the production cost of the internal oxidation method is high, and the increase in strength is accompanied by the loss of ductility and formability. The electrode is prone to cracking during extrusion forming and spot welding, and the welding performance is unstable. In addition, there are also surface strengthening methods, including brush plating, laser surface cladding, ion implantation, metal infiltration, and electrical discharge deposition [7]. However, surface coatings such as hard ceramics are easy to crack or even peel, and the service performance and life span of coated parts are not ideal.

As mentioned above, the current industrial methods of reinforcing Cu have the contradiction of a significant decrease in conductivity and ductility. Considering the superior designability and comprehensive performance of composites, one of the effective approaches to solve the problem is to prepare Cu composites with balanced mechanical properties, conductivity, and ductility by selecting appropriate reinforcements and controlling the composite structure and interface.

The physical basis of the composite effect is the property difference between the metal matrix and reinforcement. To generate obvious benefits through reinforcement, the performance of the selected reinforcements must be far superior to that of the Cu matrix. Carbon nanotubes (CNTs) have a near-perfect bonding structure and high thermal stability [8]. Their mechanical properties and axial conductivity are far better than those of a Cu matrix [9], which makes them an ideal candidate for reinforcement of Cu with little loss of conductivity and ductility of composites. Dong et al. [10] first prepared Cu matrix composites by powder metallurgy, rolling, and vacuum annealing after electroless nickel plating on CNTs in 2000. The hardness of the CNTs/Cu composite was 115–125 HV, and the conductivity was about 1/2–2/3 of pure Cu. Since then, the mechanical properties, conductivity, and ductility of CNT-reinforced Cu composites have been extensively investigated [11], mainly facing the following three challenges.

Firstly, the aspect ratio of CNTs is very high (≥1:10^3^), and van der Waals’s force and the electrostatic attraction between them result in their easy entanglement. Agglomeration of CNTs significantly reduces their strengthening effect [12] and makes it difficult to obtain uniform and stable properties of the composites. In addition, the large difference in mass density between Cu and CNTs causes mixing difficulties. However, the uniform and stable reinforcement effect can be obtained only when the reinforcements are dispersed in composites. This makes it difficult and important to study the spatial distribution of CNTs in composites [13,14,15]. At present, the dispersion methods of CNTs include mechanical ball milling [15], molecular level mixing [16], freeze-drying [17,18], ultrasonic methods [19], and dry particles impact co-mixing [20], but mechanical milling easily contaminates powders and damages CNTs. The molecular-level mixing and freeze-drying process are complicated, time-consuming, and costly. Ultrasonic & dry particle impact co-mixing is not valid for the dispersion of CNTs. In our previous work, the nitrogen bubble-assisted ultrasonic dispersion method was used, which proved to be effective to solve the problem of CNT agglomeration in composites [21,22]. This method was used in this work.

Secondly, the conductive/thermal conductive functional parts usually have higher requirements for conductivity and mechanical properties in a certain direction only. However, the excellent axial conduction properties and high aspect ratio of CNTs are not fully reflected in the macro-properties of currently prepared CNT-reinforced Cu composites owing to the random orientation of the CNT arrangement [23]. Therefore, it is necessary to spatially arrange and orient CNTs in composites along the electrical/thermal conduction direction through reasonable preparation processes and/or post-deformation treatments [24,25] to fully exploit their advantages. For this purpose, researchers tried to use magnetic fields [17] and high-ratio differential rolling (HRDSR) [26] to arrange CNTs along the axial direction, but the former did not work well, and the thermal conductivity of the composite was only 5–15% of that of pure Cu. Although the results demonstrated that high shear strain could induce the preferential dispersion and reorientation of randomly oriented CNTs along shear flow lines, HRDSR could not control the arrangement of CNTs in the rolling plane. Shuai et al. [27] prepared layered Cu/SACNT composites with an alternating arrangement of super-aligned CNT films (SACNT films) and Cu layers by layer-by-layer electrodeposition. Xiong [28] rolled them at room temperature and achieved high tensile strength (470 MPa) and high conductivity (98 IACS%) with 2.5 vol.% SACNT and 40% deformation, but the process was complicated, costly, and not efficient. The hot extrusion and cold drawing processes were adopted to ensure CNTs aligned in the matrix in our previous work, and CNTs were not damaged during the preparation process [22]. This method was used in this work.

The third challenge is interface-related. As the bond between the matrix and CNTs, the interface has the charge of transferring load, current and heat, and blocking crack extension [29]. However, the wettability of CNTs with respect to the Cu matrix is weak (wettability angle: 145°) [13], and no chemical reaction occurs between CNTs and Cu, resulting in weak bonding between CNTs and the Cu matrix. Current methods to enhance interface adhesion include the introduction of oxygen-containing functional groups, initiation of interfacial reactions, and deposition of intermediate layers. Acid treatment can introduce oxygen-containing functional groups on CNTs, forming Cu–O–C covalent bonds at subsequent high temperatures [30,31], which can improve the interface bonding. However, acid treatment tends to damage or modify CNTs [32]. Zuo et al. [29,33] added Cr, Nb, Zr, Mo, Ti, W, and other carbide-forming elements into the matrix. A carbide transition layer formed in situ at the interface through the reaction between CNTs and carbide-forming elements, which improved the wettability and interface bonding between CNTs and the Cu matrix; the result was that the composites had high conductivity and high hardness simultaneously. The sintered composite doped with W had the best performance, with a conductivity of about 96.6 IACS% and hardness of about 68.54 HV. However, the reaction between CNTs and carbide forming elements damaged or even consumed CNTs and reduced the carrying capacity of CNTs. The interfacial reaction also introduced brittle impurities, resulting in a decline in overall performance.

The metal coating deposited on CNTs by electroless plating [13,23] can be used as an intermediate layer to improve the interfacial bonding without damaging CNTs. Duan found that Ni coating could improve the interface bonding strength of CNTs/Cu composites [34]. In our previous work [22], Cu, Ni, and other coatings were electroless plated on CNTs. In the prepared Cu matrix composites, the interface between CNTs and the matrix was closely bonded, and the integrity of the CNTs was maintained, which confirmed the feasibility of improving the interface bonding by an electroless plating intermediate layer.

Among the above three problems, the dispersion and arrangement of CNTs in composites have been fully and thoroughly investigated. The composition, microstructure of the interface, and its formation mechanism, as well as their influence and mechanisms on the mechanical properties, conductivity, and ductility of composites, are still unclear. Therefore, given the contradiction between the significant decrease in conductivity and ductility caused by the current industrial methods for strengthening Cu, here Ni, Cu, and Ni&Cu coatings were deposited on multi-walled carbon nanotubes (MWCNTs) by a chemical plating process as intermediate layers to improve the interface adhesion between CNTs and the Cu matrix. Then, Cu matrix composites were prepared by hot extrusion and cold drawing after nitrogen bubbling-assisted ultrasonic dispersion. The effects of the spatially distributed and aligned orientation of MWCNTs and the composition and microstructure of the interface on the macroscopic anisotropy of composites such as mechanical properties, conductivity, and ductility were investigated. This study can provide new insights for balancing the mechanical and conductive properties of CNTs/Cu composites by controlling microstructure and interface.

## 2. Experiments

### 2.1. Materials and Preparation

Highly conductive MWCNTs (90% purity, 0.22 g/cm^3^ density, 10–20 nm diameter, 10–30 μm length) were prepared by the vapor deposition method from the Chinese Academy of Sciences Chengdu Organic Chemistry Co, Ltd. (Chengdu, China). Electrolytic Cu powder was commercially purchased with a purity of 99.8% and a size of 50–75 μm.

Ultrasonication of MWCNTs (0.4 g/L) was performed in deionized water for 10 min using a probe ultrasonic generator (20 kHz, 60 W). Subsequently, MWCNTs were added to nitric acid solution (6 mol/L) and refluxed at 115 °C–120 °C for 60 min. They were then filtered and washed until the pH value was 7. Sodium hydroxide solution (6 mol/L) and hydrochloric acid solution (6 mol/L) were added in turn and refluxed at 115 °C–120 °C for 40 min. They were then filtered and washed until the pH value was 7. The purified MWCNTs were sensitized and activated [21], followed by electroless plating with Ni, Cu, and Ni&Cu. Table 1, Table 2 and Table 3 show the formulas and conditions of the electroless plating.

In the following sections, B-CNT, Ni@CNT, Cu@CNT and Ni&Cu@CNT denote uncoated CNT, Ni-plated CNT, Cu-plated CNT, and Ni&Cu-plated CNT, respectively. B-CNTs/Cu composite, Ni@CNTs/Cu composite, Cu@CNTs/Cu composite, and Ni&Cu@CNTs/Cu composite denote the uncoated MWCNT-reinforced Cu matrix composite, Ni-plated CNT-reinforced Cu matrix composite, Cu-plated CNT-reinforced Cu matrix composite, and Ni&Cu-plated CNT-reinforced Cu matrix composite.

After electroless plating, B-CNTs, Cu@CNTs, Ni@CNTs, and Ni&Cu@CNTs were mixed with pure Cu powder (the content of MWCNTs was 3 vol.%), and Ф16 rods were prepared by cold pressing, sintering, hot extrusion, cold drawing, and turning using a previously reported process [22]. Pure Cu samples for comparison reference were prepared by the same method.

### 2.2. Characterization and Testing

The microstructure was characterized by field emission scanning electron microscopy (SEM, Apreo, FEI, Waltham, MA, USA, equipped with energy dispersive spectrometer (EDS)) and transmission electron microscopy (TEM, Talos F200X, FEI, Waltham, MA, USA). Silicon carbide (600, 800, 1000, and 1200 grit) and diamond spray polishes (W1.0 μm and W0.25 μm) were used to polish the sample surface for SEM detection. The samples were thinned to less than 100 nm using a dual-beam microscope (Scios 2, FEI, Waltham, MA, USA) and a focused ion beam (FIB) to fabricate foils for TEM detection. The surface microstructure of the samples was characterized by an electron backscatter diffraction (EBSD) detector mounted on a field emission SEM. The sample surface was electroplated at room temperature after mechanical polishing.

The tensile and compressive properties at room temperature were tested using a microcomputer-controlled electronic universal material testing machine (CTM-9200, Dongguan Hangcheng Electronic Technology, Dongguan, China) respectively. The tensile direction of specimens was parallel to the drawing direction. Specimens were processed by electrical discharge machining into a dog bone shape (16.5 mm × 6 mm × 2 mm). Compression specimens were cylindrical with a standard size of Ф18 × 25 mm and surface roughness of Ra < 0.8. Tensile tests were performed at a crosshead speed of 2 mm/min. The results are the average of five measurements.

The Keithley-2400 current source meter (Shenzhen Shijia Instrument Co., Ltd., Shenzhen, China) and Keithley-2182 voltmeter (Shenzhen Shijia Instrument Co., Ltd., Shenzhen, China) were used to test the electrical conductivity. The sample size was Φ18 mm × 5 mm. The thermal conductivity test was performed using a laser thermal conductivity analyzer (LFA427/457, NETZSCH, Selb, Germany) at 10–15 mbar vacuum. The sample size was Φ10 mm × 3 mm. The results are the average of five measurements.

## 3. Macro Performance Test Results

**Figure 1:**
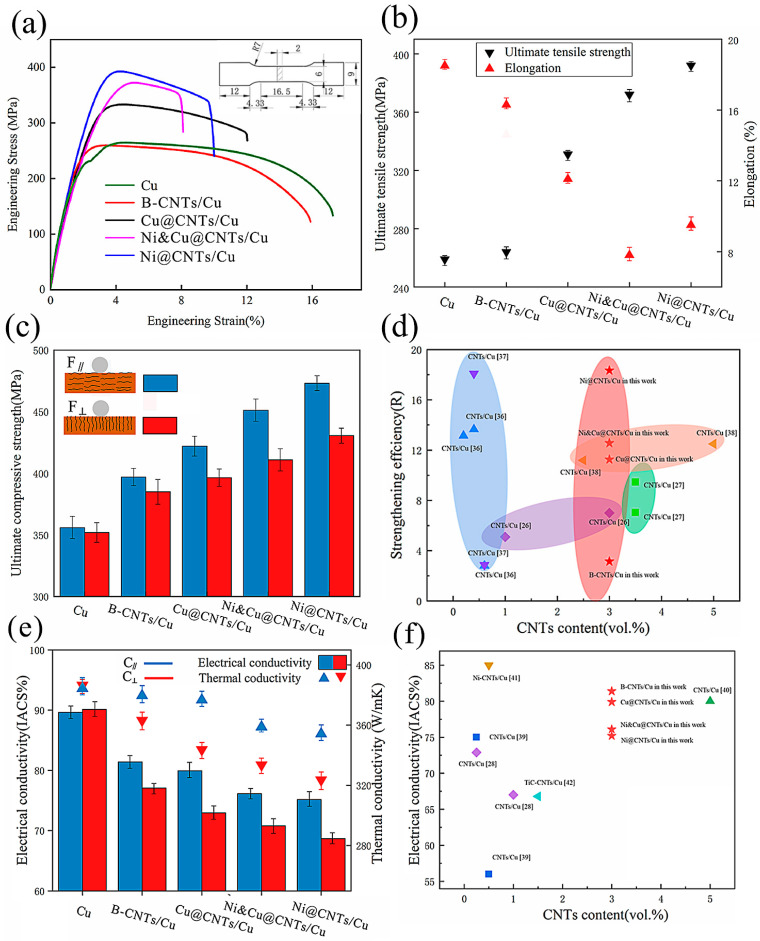
(**a**) Stress–strain curves, (**b**) ultimate tensile strength and elongation, (**c**) compressive strength of samples. (**d**) R-value of CNTs in this work compared with data in the literature [11,26,36,37,38]. (**e**) Electrical conductivity and thermal conductivity of samples. *F*_∥_, *C*_∥_ test plane parallel to the drawing direction, *F*_⊥_, *C*_⊥_ test plane perpendicular to the drawing direction. (**f**) Electrical conductivity of samples compared with the published data [28,39,40,41,42].

Figure 1 and Table 4 show the mechanical and conductivity properties of pure Cu and MWCNT-reinforced Cu matrix composite samples. The ultimate compressive strength (UTS) of pure Cu was determined according to the stress–strain (20%) of the B-CNTs/Cu composite due to the good plasticity of pure Cu, which does not have ultimate compressive strength in the test range. This indicates that coated MWCNTs significantly enhanced the Cu matrix over pure Cu samples. The enhancement effect of B-CNTs on the Cu matrix was far less than that of coated MWCNTs. The tensile strength increased slightly, and the compressive strength increased significantly. Our previous study [22] demonstrated that the density of 3 vol.% MWCNT-reinforced Cu matrix composite made by the same process was close to full density, reaching 99.67%. The effect of porosity on material properties can be ignored. Therefore, the above results indicate that the coating had a strong influence on interface bonding and mechanical properties. Among them, the Ni@CNTs/Cu composite had the greatest effect, and its Ultimate tensile strength (UTS), Yield Strength (YS), and Ultimate compressive strength (UCS) reached 391 MPa, 358 MPa, and 473 MPa, respectively, i.e., 50.97%, 54.98%, and 32.86% higher than those of pure Cu sample (259 MPa, 231 MPa, and 356 MPa). The Ni&Cu@CNTs/Cu composite had the next greatest effect, followed by the Cu@CNTs/Cu composite. In order to assess the strengthening contribution of CNTs, the strengthening efficiency (R) of CNTs was determined according to the following formula [35]:

(1) $$R=\frac{\sigma_{c}-\sigma_{m}}{\sigma_{m}V_{\mathrm{CNT}}}$$

where $\sigma_{c}$ is the yield strength of the composite,${\;\sigma }_{m}$ is the yield strength of the Cu matrix, and $\sigma_{m}$ is the volume fraction of CNTs. The R-value of CNTs in this work compared with data in the literature [11,26,36,37,38] is shown in Figure 1d. It demonstrates that the strengthening efficiency of CNTs in our work is higher than that published in most literature.

The elongation of composite samples decreased. The elongation from high to low was as follows: B-CNTs/Cu composite, Cu@CNTs/Cu composite, Ni@CNTs/Cu composite, and Ni&Cu@CNTs/Cu composite. Among them, Ni&Cu@CNTs/Cu decreased from 18.5% of the pure Cu sample to 7.5%.

Compared to pure Cu samples, the conductivity of composite samples also decreased, as can be seen in Figure 1e. From high to low, the ranking was as follows: B-CNTs/Cu composite, Cu@CNTs/Cu composite, Ni&Cu@CNTs/Cu composite, and Ni@CNTs/Cu composite, which is opposite to the pattern of the strength increase in the composites. Interestingly, the B-CNTs/Cu composite showed the smallest decrease in conductivity. The electrical conductivity of the composites in this work compared with the published data [28,39,40,41,42] is shown in Figure 1f. It was also higher than that reported in most literature.

It can be seen from Figure 1c,e that the composite samples exhibited orthogonal anisotropy in both mechanical and conductivity properties. The strength and conductivity in the direction parallel to the drawing direction were significantly higher than those in the vertical direction. In practical applications, the directions of heat/current flow or load should be consistent with the direction of optimal material performance. Therefore, we are more concerned about the properties along with the F_∥_ and C_∥_ directions (parallel to the drawing direction). The experimental data demonstrated that the comprehensive performance of the Cu@CNTs/Cu composite was better, and its enhancement was high, while the ductility and conductivity were not excessively reduced.

## 4. Microscopic Characterization Results

### 4.1. Coated MWCNTs

**Figure 2:**
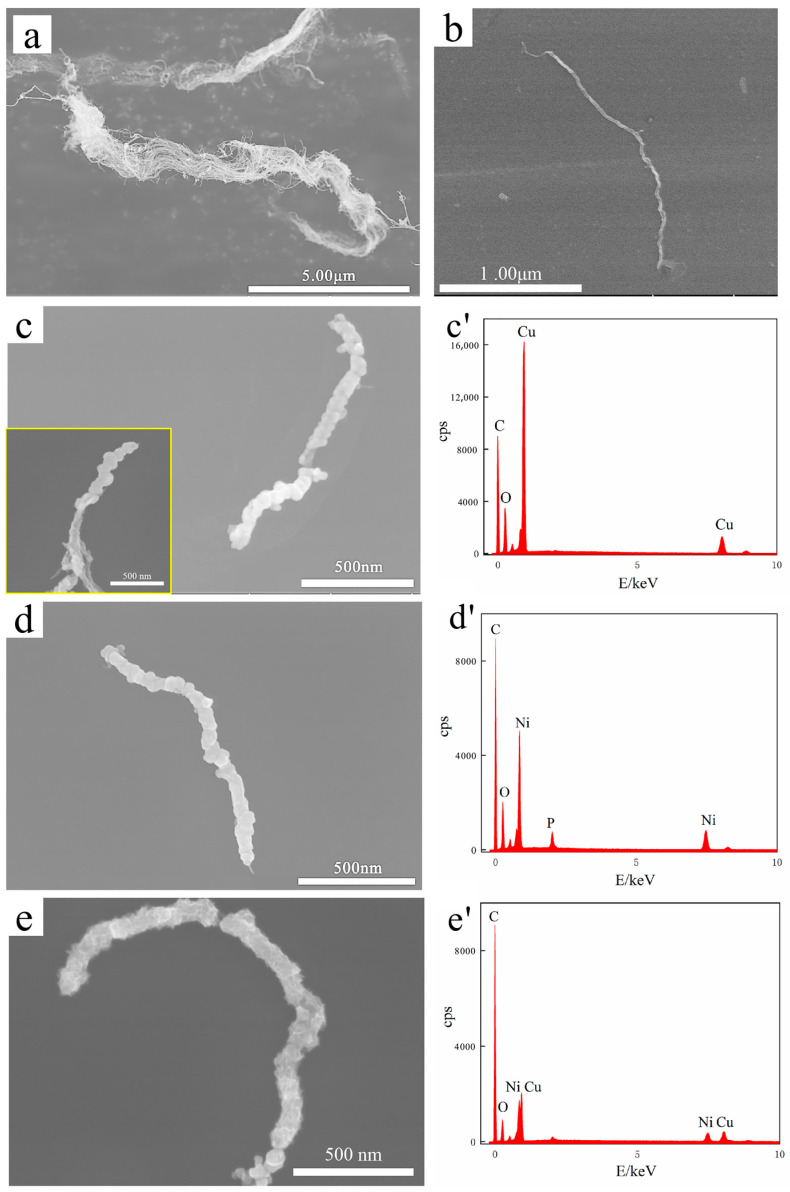
SEM images and EDS analysis of (**a**) pristine MWCNTs, (**b**) MWCNTs after ultrasonic treatment, (**c**) Cu@CNT (The illustration is Cu@CNT at different positions.), (**d**) Ni@CNT, (**e**) Ni&Cu@CNT. (**c’**–**e’**) are EDS analyses of the corresponding sample surface.

Figure 2 displays the SEM images and EDS analysis of the original MWCNTs and MWCNTs before and after electroless plating. The original MWCNTs were bundled with tangles ≥ 1 μm in diameter, as shown in Figure 2a. After ultrasonic treatment, MWCNTs were untangled into single roots with a diameter of 10–20 nm and length of ~2 μm, as shown in Figure 2b. Figure 2c–e shows Cu@CNT, Ni&Cu@CNT, and Ni@CNT, respectively, with diameter > 100 nm and length > 1μm, maintaining a large aspect ratio. Among them, Ni@CNT had the most dense, continuous, and complete coating. In contrast, the coating on Cu@CNT and Ni&Cu@CNT was loose and uneven, and there were uncoated parts on MWCNTs (Figure 2c). This is because Ni has self-catalytic activity for electroless plating, which can effectively be replaced with Pd catalyst particles that adhere to MWCNTs and reduce and deposit on MWCNTs. In addition, after the Pd catalyst is totally consumed, the catalytic Ni surface can continue the deposition reaction to fill the uncoated gap or increase the thickness of the coating. Therefore, Ni plating was the most dense, continuous, and complete. Copper has no catalytic activity, and its potential was positive than that of Ni. Once the Pd catalyst was completely consumed, the deposition reaction stopped immediately, and the uncoated gap could not be filled. The reason for incomplete and uniform Ni&Cu coating is that the main salt in the plating bath was mostly CuSO_4_, and the NiSO_4_ content was much lower than that of the Ni-plating bath, which was not enough to form a complete coating.

Furthermore, EDS elemental analysis in Figure 2c’–e’ demonstrates that all three coatings contained oxygen. Our previous study [43] demonstrated that the oxidation of the Cu coating was much more serious than that of the Ni coating, which was almost unoxidized. Therefore, the oxygen element detected by EDS analysis in Ni coating here was not necessarily oxidized during electroless plating and powder mixing. It is also possible that oxygen was introduced into the sample storage and SEM sample preparation.

### 4.2. Orientation of MWCNTs in Composites

**Figure 3:**
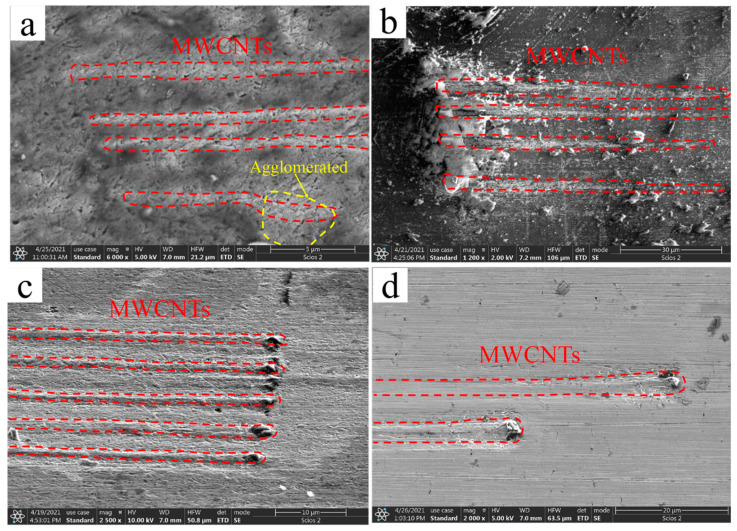
SEM images of (**a**) MWCNTs/Cu composite, (**b**) Cu@CNTs/Cu composite, (**c**) Ni@CNTs/Cu composite, (**d**) Ni&Cu@CNTs/Cu composite.

Figure 3 demonstrates the representative SEM images of the polished composite sample’s surface. Many parallel elongated rod-like protrusions were found on each sample, some of which were even longer than 70 μm. The three-dimensional TEM thin slices were extracted by femtosecond laser with a dual-beam microscope, and the thickness of the slices enveloped these protrusions. The TEM detection plane on both sides of the thin slices was parallel to the length direction of the rod-like protrusions. It can be deduced from the TEM that these rods were metal-coated MWCNTs. Figure 3 shows that these MWCNTs protrusions were straight and long. They were separated from each other and maintained a highly consistent alignment. This is strong evidence that our process can realize the highly oriented arrangement of MWCNTs in the Cu matrix. However, there are some bumps on the B-CNTs/Cu composite surface that were not straight enough and even had agglomerates. This may be because bare MWCNTs have poor stiffness and are more prone to deformation and entanglements compared with coated CNTs.

**Figure 4:**
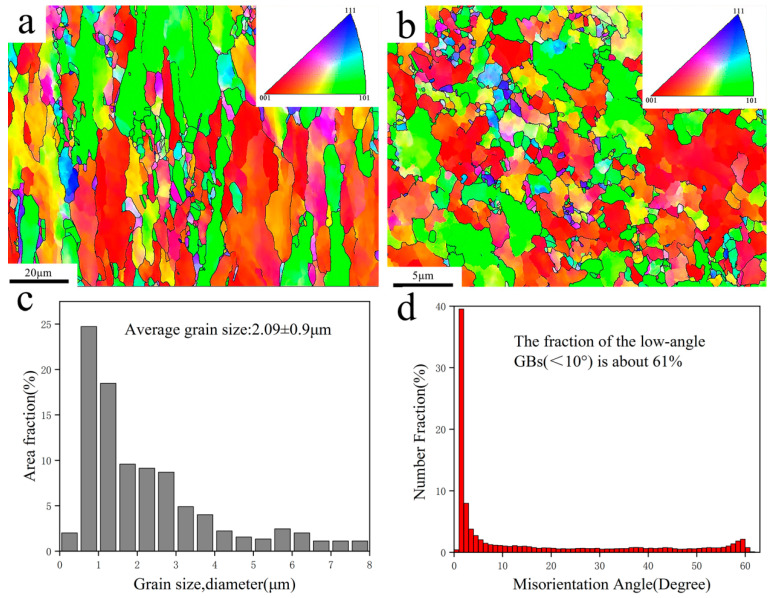
EBSD images of Ni@CNTs/Cu composites: (**a**) test plane parallel to the drawing direction, (**b**) test plane perpendicular to the drawing direction. (**c**) Grain size distribution of (**a**,**d**) Grain boundary orientation difference distribution of (**a**).

To further understand the microstructure of the orientation, EBSD analysis was carried out on four composite samples. Taking the EBSD images of the Ni@CNTs/Cu composite in the plane parallel to the drawing direction and that vertical to the drawing direction shown in Figure 4 as an example, the Ni@CNTs/Cu composite had a strong (001) oriented texture in the plane parallel to the drawing direction (as demonstrated in Figure 4a). Compared with that vertical to the drawing direction (as demonstrated in Figure 4b), the grains were significantly elongated. This is mainly because the Cu grains lengthened along the maximum deformation direction and compressed in the radial direction under the bidirectional compressive stress generated during the severe deformation of hot extrusion and drawing. A large amount of compressive stress and shear strain were generated during this process, which also forced the MWCNTs to be highly oriented and straightened along the drawing direction.

The results of EBSD analysis in Table 5 also indicate that the average grain sizes of the composites from large to small were as follows: B-CNTs/Cu composite, Cu@CNTs/Cu composite, Ni&Cu@CNTs/Cu composite, and Ni@CNTs/Cu composite (3.01 (±0.95) μm, 2.37 (±1.03) μm, 2.21 (±0.97) μm, 2.09 (±0.9) μm), respectively), which were significantly smaller than the average grain size of pure Cu (3.32 (±1.1) μm). Our previous study [22] demonstrated that MWCNTs are homodispersed in composites. These homodispersed MWCNTs had a pinning effect on Cu grain boundaries (GBs) [44,45], which could effectively reduce or restrict the Cu grain growth of the matrix at high temperatures. In addition, taking the Ni@CNTs/Cu composites shown in Figure 4d and Table 5 as an example, the proportion of low-angle GBs (<10°) was as high as 61%, which was the highest among the four composites in this work. The grain boundary energy mainly originates from the dislocation energy, which is determined by the phasic diversity between grains. Therefore, the relationship between the grain boundary energy and orientation difference conforms to the formula [46]:

(2) $$\gamma =\frac{Gb}{4\pi \left(1-v\right)}\cdot \theta \left(A-Ln\theta \right)$$

where G is the shear modulus of the composite, b is Burgers vectors, v is Poisson’s ratio, θ is a phase difference, and A is an integration constant that is determined by the atomic dislocation energy at the dislocation center. Formula (2) indicates that the interface energy of small-angle GBs was lower; hence, the interface was more steady. In the composites, the higher the content of low-angle GBs, the stronger the bonding between matrix grains. Low-angle GBs can also improve the ductility of metal for dislocations that are prone to shift through adjacent grains with slightly disoriented lattices [9]. The above data also revealed that the mechanical properties of the Ni@CNTs/Cu composite were the best among the four composites. One of the reasons is the lowest interfacial energy and more stable interface of the Ni@CNTs/Cu composite.

In this work, a highly oriented arrangement of CNTs in the Cu matrix was achieved. In practical applications, it is beneficial to give full play to the excellent axial performance of CNTs to make the working load direction consistent with this direction.

### 4.3. MWCNTs/Cu Interface

**Figure 5:**
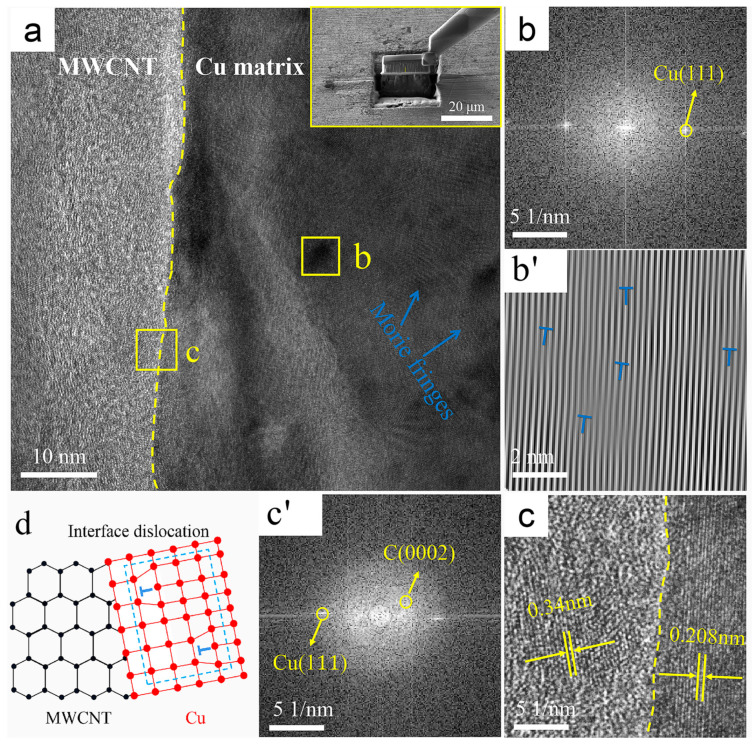
TEM images of the B-CNTs/Cu composite: (**a**) interface, upper right corner illustration shows manipulator sampling (Illustration is TEM sampling location); (**b**) FFT image of region b in (**a**); (**b’**) IFFT image of region b in (**a**), with dislocations marked by “T” symbols; (**c**) enlarged view of region c in (**a**), and (**c’**) is its FFT image; (**d**) schematic of interface dislocations.

Figure 5 shows representative TEM images of the interface region between MWCNTs and the Cu matrix in the B-CNTs/Cu composite and the corresponding fast Fourier transform (FFT) and inverse fast Fourier transform (IFFT). Through the characteristic atomic arrangement, two regions were identified in Figure 5c’ based on FFT patterns, namely, in Figure 5a,c, the left side of the yellow dotted line was the graphite structure C (0002) lattice of CNTs, and the right side was the lattice Cu (111). No gaps and other defects were found between MWCNTs and the matrix, indicating a tight contact between Cu and CNTs. Figure 5c also reveals a well-defined boundary at the interface, with an abrupt transition from a C(0002) plane with 0.34 nm planar spacing of MWCNTs on the left side of the yellow dotted line to a Cu(111) plane with 0.208 nm planar spacing of Cu on the right side, and no transition region was found between them. Comparing the crystallographic orientation relationship between them, it was found that they were not parallel. These are typical mechanically bonded interface features. This is determined by the limited wettability of MWCNTs with respect to the Cu matrix [13].

There were some darker colored regions to the right of the yellow dotted line where clear Moire fringes were visible, as shown in region b in Figure 5a. The FFT and IFFT plots of this region (dislocations are marked with “T” symbols) are shown in Figure 5b,b’, and the Moire fringes revealed that there were dislocations distributed among them. This indicates that large compressive stresses and shear strains were generated near MWCNTs during the severe deformation process of hot extrusion and drawing, resulting in the formation of dislocations. The schematic diagram of this dislocation formation is shown in Figure 5d.

**Figure 6:**
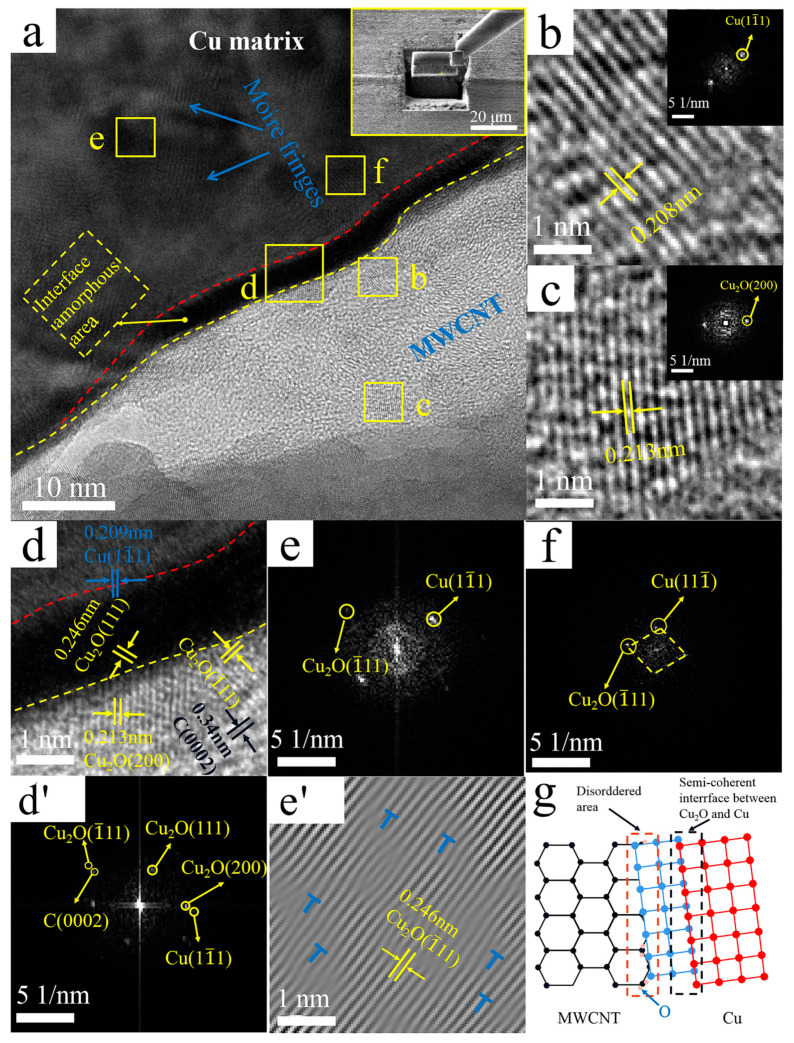
TEM images of the Cu@CNTs/Cu composite: (**a**) interface, upper right corner illustration shows manipulator sampling (Illustration is TEM sampling location); (**b**) enlarged view of region b in (**a**) (The illustration is the corresponding FFT image); (**c**) enlarged view of region c in (**a**) (The illustration is the corresponding FFT image); (**d**,**d’**) enlarged views and FFT image of region d in (**a**); (**e**,**e’**) FFT image and IFFT image of region e in (**a**), with dislocations marked by “T” symbols; (**f**) FFT image of region f in (**a**); (**g**) interface schematic.

Figure 6 shows representative TEM images of the interface region of Cu@CNTs/Cu composites and the corresponding FFT and IFFT images. Through measurement of the crystal plane spacing in combination with the FFT patterns, three zones were recognized in Figure 6d’ The plane spacing below the yellow dotted line in Figure 6a,d was ~0.34 nm, which corresponds to the C(0002) lattice of MWCNTs. The crystal plane spacing above the red dotted line was ~0.209 nm, which corresponds to the Cu(1$\bar{1}$1) plane; the plane spacing between red and yellow dotted lines was ~0.246 nm, which corresponds to the Cu_2_O(111) plane. In addition, on the lower side of the yellow dashed line, region b, and region c in Figure 6d, we detected an interplanar spacing of ~0.213 nm, ~0.208 nm, and ~0.213 nm, corresponding to the Cu_2_O(200) plane, Cu($\bar{1}$11) plane, and Cu_2_O(200) plane, respectively, and (0002)_MWCNT_//($\bar{1}$11)_Cu_, demonstrating that the Cu was grown in situ at the interface. Therefore, it was determined that this Cu on MWCNTs originated from chemical plating. As for Cu_2_O, it may originate from a variety of factors: (1) oxidation of the electroless Cu coating. The oxidation may occur during the composite preparation process, such as storage, powder mixing, and sintering. (2) Cu reacted with oxygen-containing functional groups to form Cu_2_O. The acid treatment used in this work may have introduced oxygen-containing functional groups, e.g., carboxyl and hydroxyl groups on MWCNTs [47], which are prone to react with Cu. The schematic diagram of the MWCNTs-Cu_2_O-Cu interface is shown in Figure 6g. Studies have demonstrated that MWCNTs/Cu_2_O is a gradual transition from the helical lattice plane of Cu_2_O to the C(0002) lattice of MWCNT, and Cu_2_O at the Cu@CNTs/Cu interface plays an important role in regulating the transition medium between MWCNTs and Cu matrix [48]. Therefore, the electroless Cu coating and Cu_2_O nanocrystals that formed in situ at interfaces can tightly connect MWCNTs and matrix, forming strong chemical bonding, which is beneficial to improve the interfacial combining strength and load transfer efficiency [30,49,50,51]. Compared with the interface in the B-CNTs/Cu composite, the amorphous transition zone with a thickness of 6–8 nm formed at the interface along the sidewall of MWCNTs, as shown between the red dashed line and yellow dotted line in Figure 6a,d. There were no gaps and other defects between MWCNTs, the amorphous transition zone, and Cu matrix, indicating close interfacial bonding in the Cu@CNTs/Cu composite.

From the FFT diagram of the amorphous transition region in Figure 6d’, we identified that the crystal diffraction pattern corresponded to the Cu(1$\bar{1}$1) plane and Cu_2_O(1$\bar{1}$1) plane and (1$\bar{1}$1) _Cu_//(1$\bar{1}$1) _Cu2O_ (as shown in Figure 6d’). Similarly, in the matrix near the amorphous transition region, the FFT diagram of region f in Figure 6a,f also identified the Cu(11$\bar{1}$) plane and Cu_2_O ($\bar{1}$11) plane and (11$\bar{1}$) _Cu_//($\bar{1}$11) _Cu2O_ It indicated that the coherence interface formed between Cu and Cu_2_O through the parallel crystal plane, resulting in a higher coordinated deformability of MWCNTs and Cu matrix under loading, which facilitated a fine balance between the strength and ductility of the composites.

There were some darker regions in the matrix in Figure 6a, and clear Moire fringes are shown. Figure 6e,e’ show FFT and IFFT images of region e in Figure 6a, and the Moire fringes revealed that the dislocation densities were greater than those in the matrix of the B-CNTs/Cu composite. The reasons include (1) High compressive stress and shear strain generated by severe deformation of extrusion and drawing. (2) Low-angle GBs formed at the interface by thermal mismatch between MWCNTs and the Cu matrix, which generated internal stresses at the interface. New dislocations might also be generated at the interface during rapid cooling after hot extrusion, thus densifying the dislocations [52]. (3) Distortion of the crystal lattice caused by the different parameters of the crystal lattice of Cu and Cu_2_O in the matrix and the coatings. The interaction between dislocation stress fields could impede dislocation motion by mutual repulsion or attraction [53]. The increase in dislocation density enlarged the interfacial shear stress, thus enhancing the interfacial bonding between CNTs and the matrix.

**Figure 7:**
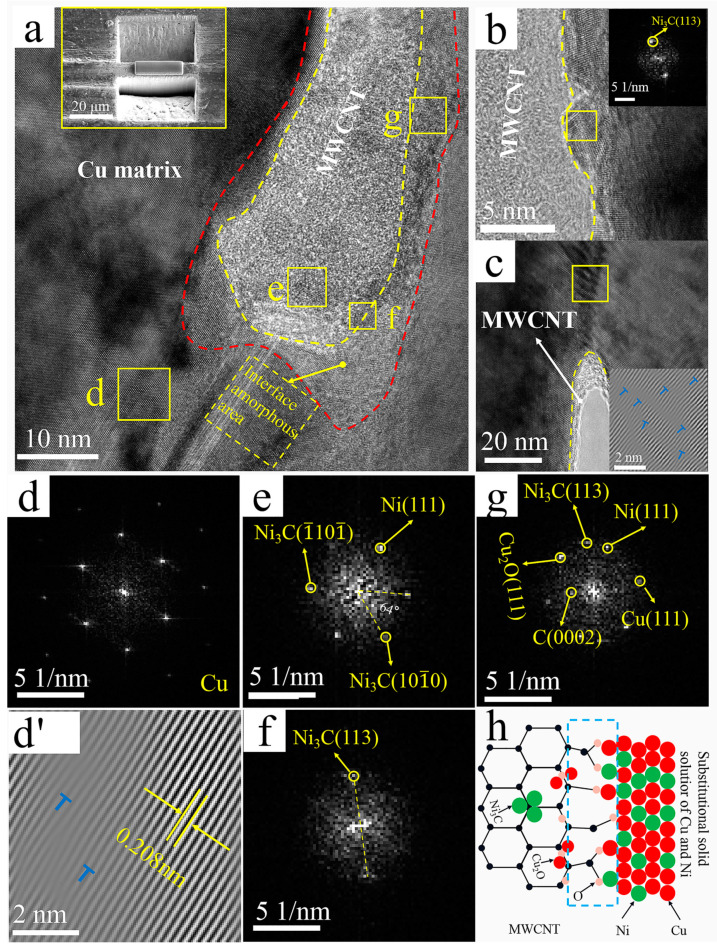
TEM images of the Ni@CNTs/Cu composite: (**a**) interface, top left inset shows manipulator sampling (Illustration is TEM sampling location); (**b**) Interface between MWCNT and Cu (Illustration is an FFT image of the yellow area); (**c**) Interface between MWCNT and Cu (Illustration is an IFFT image of the yellow area); (**d**,**d’**) FFT and IFFT images of region d in a, with dislocations marked by “T” symbols; (**e**–**g**) FFT images of the corresponding regions in (**a**); (**h**) Schematic diagram of interface formation.

Figure 7 shows the high-resolution TEM images of the interface region of the Ni@CNTs/Cu composite, as well as the corresponding FFT and IFFT images. Three regions were recognized in Figure 7a: MWCNTs in the middle of yellow dashed lines, amorphous transition regions between red and yellow dashed lines, and Cu matrix outside the red dashed line. Moreover, the Ni(111) plane, Ni_3_C(113) plane, Ni_3_C($\bar{1}$10$\bar{1}$) plane, and Ni_3_C( 10$\bar{1}$0) plane were identified on MWCNTs by crystal plane spacing according to FFT patterns, as shown in Figure 7b,e,f. The Ni on the MWCNTs originated from electroless Ni coating. The metastable hexagonal nanocrystalline Ni_3_C might be formed by the reaction of electroless Ni plating with MWCNTs during sintering and/or extrusion. Chen et al. [54] reported that carbides preferentially grew in situ at the defects on the CNT surface. Therefore, the Ni_3_C was not uniformly distributed but tended to form defect sites on MWCNT. These defect sites mostly originated from the acid treatment used in this work, which introduced oxygen-containing functional groups on MWCNTs.

**Figure 8:**
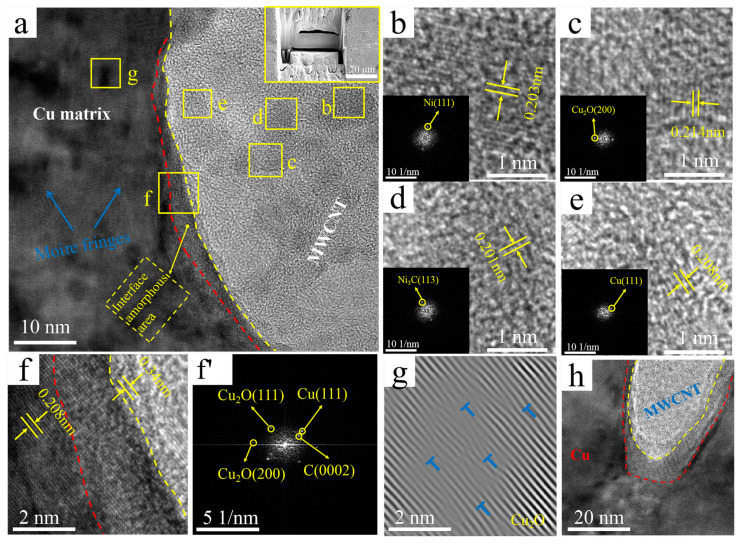
TEM images of the Ni&Cu@CNTs/Cu composite: (**a**) interface, upper right inset shows manipulator sampling (Illustration is TEM sampling location); (**b**–**e**) the enlarged image of the corresponding region in (**a**) (The illustration is the corresponding FFT image); (**f**,**f’**) enlarged and IFFT images of region in (**a**); (**g**) IFFT image of region g in (**a**), with dislocations marked by “T” symbols; (**h**) interface at the end of MWCNT.

Similar to the Cu@CNTs/Cu composite, a 6–8-nm-thick amorphous transition zone along the sidewalls of MWCNTs was formed at the interface, as shown in the regions between the red and yellow dashed lines in Figure 7b and Figure 8a. The Ni(111) plane, Ni_3_C(113), planar Cu(111) plane, and Cu_2_O(111) plane were identified according to the FFT patterns of amorphous transition regions in Figure 7b,g. No defects such as gaps were found in the interfacial region. The amorphous transition region was from the graphite structure of MWCNTs to the clear arrangement of Ni atoms and then to the fuzzy Ni lattice planes, indicating that the Ni@CNTs/Cu composite formed close interface bonding. The amorphous transition zones not only provided the source sites for dislocation initiation but also served as high-capacity zones for dislocation pinning and stacking during deformation, resulting in the interface with a high load-bearing capacity [13]. Since Ni and Cu have the same face-centered cubic crystal structure and similar thermo-physical performance, Cu and Ni form an infinite solid solution; therefore, the interfacial zones were mainly composed of a solid solution of Cu and Ni, and the d orbital of Ni and the p orbital of MWCNTs could be hybridized, so there was strong chemisorption and interfacial bonding between Ni and MWCNTs. Furthermore, the source of Ni_3_C in the amorphous transition zones was the same as described before. Cu_2_O in the amorphous transition regions was also associated with defective sites of oxygen-containing functional groups on MWCNTs. The oxygen-mediated bonding of Cu-O-C contributed to the formation of Cu_2_O, which is consistent with Zhao’s s report [55], which shows a schematic diagram of Ni@CNTs/Cu interfacial bonding. [56] demonstrated that when CNTs react with a metal matrix to form oxide or carbide transition layers, the wetting angle between CNTs and the metal matrix can be reduced and interfacial bonding can be enhanced.

The slip bands are visible in the lower right of region d of Figure 7a, indicating the history of plastic deformation here. Figure 7d,d’ show the FFT and IFFT images of region d in Figure 7a. The inset in the lower right corner of Figure 7c shows the IFFT image of the region in the yellow box. These Cu GBs are mainly in an unbalanced state with external (non-geometrically necessary) dislocations. Furthermore, Moire fringes indicated that the dislocation density in Figure 7c was higher than that in Figure 7d’. The dislocation congestion in Figure 7c was caused by the accumulation of new dislocations caused by severe plastic deformation during extrusion and drawing. The plastic deformation led to the connection and diffusion of dislocation entanglement in Figure 7d’, which is a typical feature of multi-slip deformation [57].

Figure 8 demonstrates TEM images and the corresponding TTF and IFFT images of the interface regions of the Ni&Cu@CNTs/Cu composite. They have many similar characteristics with the interface of Ni@CNTs/Cu and Cu@CNTs/Cu. Three regions are also identified in Figure 8a: MWCNTs on the right side of the yellow dotted line, amorphous transition regions between red and yellow dotted lines, Cu matrix on the left side of the red line. In addition, the Ni(111) plane, Cu(111) plane, Ni_3_C(113) plane, and Cu_2_O(200) plane were identified on MWCNTs, as shown in Figure 8b–e. These Ni and Cu on MWCNTs originated from electroless plating. The sources of Ni_3_C and Cu_2_O were the same as above.

Similar to the first two composites, -6-nm-thick amorphous transition regions formed along the MWCNT side walls at the interface, as shown in the zone between the red and yellow dotted lines in Figure 8a,f. No defects such as gaps were found, demonstrating that close interfacial bonding formed in the Ni&Cu@CNTs/Cu composite. The Cu(111) plane, Cu_2_O(200) plane, and Cu_2_O(111) plane was identified from the FFT images of the amorphous transition regions in Figure 8f’, indicating that the reaction of Cu atoms with the carboxyl and hydroxyl groups on MWCNTs led to a strong MWCNTs-Cu_2_O-Cu interface. The Ni&Cu@CNTs/Cu interface had the characteristics of both Ni @CNTs/Cu and Cu@CNTs/Cu interfaces. In addition to the formation of Ni and Cu solid solutions, there were also CNT-Cu_2_O-Cu interfaces at the interface. Nevertheless, since most of the plating was Cu, the content of Ni atoms was too small, and the influence of Cu on the interface played a dominant role.

There were also some darker areas in the matrix in Figure 8a, and the clear Moire fringes are shown. The formation of Moire fringes was due to high-density dislocations. The Moire stripe in Figure 8g reveals its dislocation density higher than that in the matrix of the B-CNTs/Cu composite. Slip bands and dislocation congestions were also not found at the interface regions of the MWCNTs ends (Figure 8h).

### 4.4. Tensile Fracture

**Figure 9:**
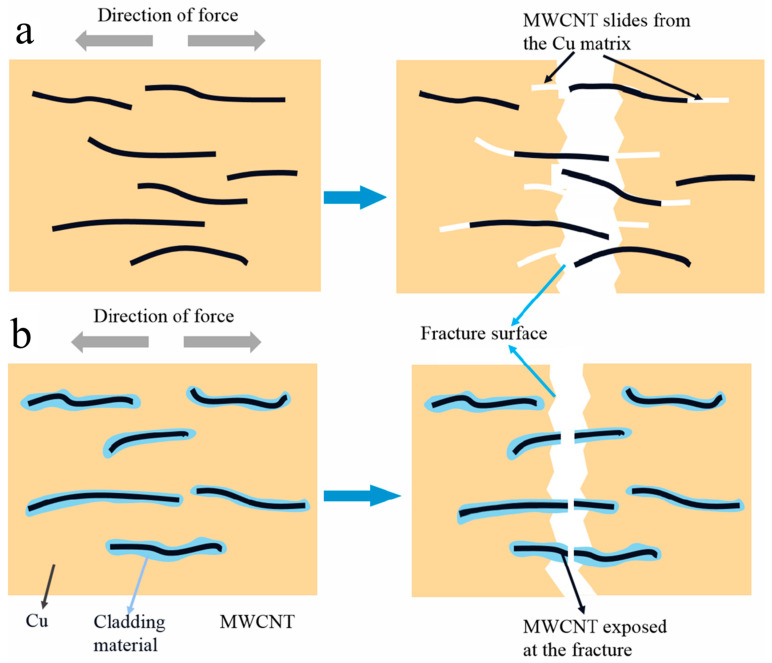
Schematic diagram of the tension fracture of (**a**) B-CNTs and (**b**) the coated MWCNT-reinforced copper composite.

It was found in our previous study [22] that there are long MWCNTs at the tensile fracture of the B-CNTs/Cu composite, indicating that MWCNTs slipped out of the Cu matrix under force, which is evidence of weak interfacial bonding. The MWCNTs exposed at the tensile fracture of coated MWCNTs reinforced copper matrix composites are short and stubby, demonstrating that the interface between MWCNTs and the matrix was tightly bonded, and MWCNTs break under greater load during tensile, instead of slipping out of the Cu matrix. Figure 9a,b are schematic diagrams of fracture modes of B-MWCNTs and coated MWCNTs during tensile respectively. Figure 9a,b show schematic diagrams of the fracture modes of the respective B-CNTs and coated MWCNTs during tension. MWCNTs have firm interfacial adhesion and inherent physical properties, which contribute to increasing the peeling length and tensile displacement between the Cu matrix and MWCNTs [58]. Through improvement of the interfacial shear stress, the inherent strength of MWCNTs along the arrangement direction was fully reflected [31]. This is consistent with reports by Wang [58] and Maqbool [59].

## 5. Strengthening Mechanism

To explore the strengthening mechanism of CNTs on composites, the reinforcement effect of CNTs was quantitatively analyzed. It mainly consists of the following aspects: (a) refinement of grains by the pinning effect of CNTs [44], (b) Orowan strengthening of CNTs, (c) load-transmitting effect, and (d) thermal mismatch mechanism of dislocations on account of different coefficients of thermal expansion (CTE) between the Cu matrix and MWCNTs. The grain refinement strengthening effect by the pinning effect of CNTs is calculated by the Hall–Petch equation [60]

(3) $$\Delta \sigma_{GR}=K\left(d_{c}{}^{-\frac{1}{2}}-d_{o}{}^{-\frac{1}{2}}\right)$$

where *K* is the constant for Cu (0.14 MPa/m^1/2^ [61]), and *d_c_* and *d_o_* are the average grain sizes of the composites and pure Cu, respectively. Orowan strengthening of CNTs can be determined by the Orowan–Ashby equation [60,62]:

(4) $$\sigma_{\mathrm{Orowan}}=\frac{0.4\mathrm{MGb}}{\pi \lambda {\left(1-v\right)}^{\frac{1}{2}}}\mathrm{In}(\frac{{\pi d}_{p}}{4b})$$

where M is the Taylor factor (3.06 for Cu [63]); λ is the effective spacing between adjacent CNTs ($\lambda =d_{p}\left(\sqrt{\frac{\pi }{2V_{CNT}}}-1\right)$); $V_{CNT}$ is the volume fraction of CNTs; *b* (0.256 nm) is the Burgers value of the matrix; $d_{p}$ is the diameter of the effective reinforcement, in which case the reinforcements are cylindrical CNTs ($d_{p}=\sqrt[{3}]{\frac{3Sl_{CNT}}{4\pi }}$), *S* is the cross-sectional area [62], $l_{CNT}$ (~6 μm) is the average length of CNTs; *G* is the shear modulus of the matrix; E (110 GPa) and v (0.355) are Young’s modulus and Poisson’s ratio of the matrix, respectively. The strengthening effect from load transfer can be given by a modified shear lag model with the equations [62,64]:

(5) $$\sigma_{L.T}=\sigma_{m}\left(1-V_{CNT}\right)+\sigma_{CNT}V_{CNT}\left(\frac{l_{CNT}}{2l_{c}}\right)\;\;\;\;for\;l_{CNT}<l_{c}$$

(6) $$\sigma_{L.T}=\sigma_{m}\left(1-V_{CNT}\right)+\sigma_{m}V_{CNT}\left(1-\frac{l_{CNT}}{2l_{c}}\right)\;for\;l_{CNT}>l_{c}$$

where $\sigma_{L.T}$ is the strength increase by load transfer, $\sigma_{m}$ and $\sigma_{CNT}$ are the yield strengths of the Cu matrix and MWCNT (~39 GPa), respectively. The critical length $l_{c}=\frac{\sigma_{CNT}d}{2\tau_{m}}$, d is the diameter of MWCNTs (~16 nm); $\tau_{m}$ is the yield shear strength of matrix ($\tau_{m}=\frac{1}{2}\sigma_{m}$). The increase in dislocation density produced by thermal mismatch makes for the strengthening of the composites, and the reinforcement effect can be derived from equation [65]:

(7) $$\sigma_{T.M}=\alpha Gb\sqrt{\frac{12V_{CNT}\Delta CTE\Delta T}{bd_{p}}}$$

where α (1.25) is a constant; ΔCTE (2.84 × 10^−5^ K^−1^ [66,67]) is the CTE mismatch between Cu and CNTs; ΔT (1103 K) is the maximum temperature change in mechanical thermal processing.

**Figure 10:**
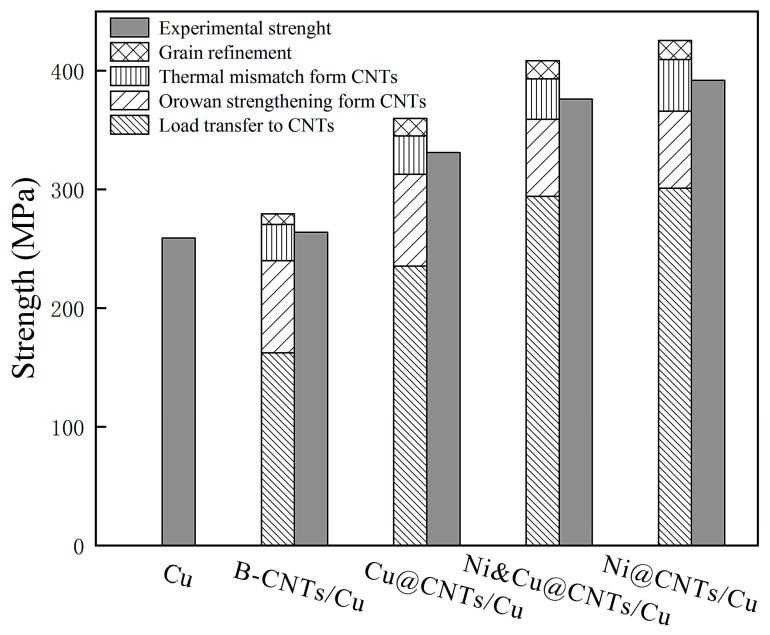
Theoretical and experimental results of the contribution of reinforcing mechanisms to tensile strength.

Figure 10 reveals the contribution of various reinforcement effects to tensile strength and the comparison of theoretical calculations and experimental results. The results indicated that the load transmitting effect was the main reinforcing mechanism in MWCNT-reinforced Cu matrix composites. The load transfer effect accounted for 72.29% of the total in Ni@CNTs/Cu composite. There was thermal expansion coefficient (CTE) mismatch between MWCNTs and the Cu matrix (CNTs is 2.1 × 10^−5^ k^−1^ [68], Cu is 1.7 × 10^−6^ k^−1^ [69]). Many new dislocations were produced during sintering, hot extrusion, and drawing. MWCNTs embedded in the matrix and the produced oxide carbides blocked the shift of dislocations, resulting in the accumulation of dislocations and the formation of Orowan rings. Orowan strengthening did not contribute much to the enhancement of the matrix, accounting for only 16.58% of the total for Ni@CNTs/Cu composite. The contribution of grain refinement caused by the pinning effect of CNTs was almost negligible.

The results of this study showed that the tensile strength of composites from high to low was the Ni@CNTs/Cu composite, Ni&Cu@CNTs/Cu composite, Cu@CNTs/Cu composite, and B-CNTs/Cu composite. Nevertheless, the enhancement of the Cu matrix by B-CNTs was far less than that by coated MWCNTs, and the tensile strength increased slightly. This is because the poor wettability of Cu and MWCNTs (wettability angle 145°) [13] determines the weak interfacial bonding. The interface between CNTs and the matrix was weakly mechanically bonded, as shown in Figure 5. When the B-CNTs/Cu composite was subjected to tensile load, the load could not effectively be transferred through the interface, and MWCNTs easily slipped out of the Cu matrix and unloaded prematurely. The metal coating on MWCNTs helped to form a tight interfacial bonding between CNTs and the matrix. At the same time, the coating is conducive to overcoming the attraction between MWCNTs and dispersing MWCNTs in the matrix. The performance of composites depends largely on the interfacial adhesion strength. Strong interfacial bonding leads to the good performance of CNTs [23,46,70].

The Ni@CNTs/Cu composite was the strongest due to the following reasons: (1) Ni plating was the densest, continuous, and complete. In contrast, the Cu and Ni&Cu coatings were looser and incomplete, as shown in Figure 2. (2) Their interfacial regions were mainly composed of solid solutions of Cu and Ni or Ni_3_C, as shown in Figure 7, with strong chemisorption and interfacial bonding. (3) The Ni@CNTs/Cu composite had a high percentage of low-angle GBs (<10°) of 61%, as shown in Table 5, which was the highest among the four composites, so the matrix intergranular bonding was the strongest. The interfacial energy of small-angle GBs was lower, and thus their interfaces were more stable. (4) High-density dislocations were generated in and around interfacial regions. These dislocations mitigated the crystal lattice deformation caused by the different parameters of the crystal lattice of Cu and Cu_2_O. The small amount of Cu_2_O at the interface had a pinning effect on GBs, thus creating resistance to their movement and reducing the interfacial migration rate, which strengthened the coated MWCNT-reinforced Cu matrix composites.

Although B-CNTs slightly affected the tensile strength of the matrix, the compressive strength improved significantly. This is because the compressive load did not cause the MWCNTs to slip out of the Cu matrix, allowing more MWCNTs to bear the load. The inherent properties of the MWCNTs (Young’s modulus reached 270–950 GPa) and the metal coating on MWCNTs can resist deformation. The force effectively was shifted from the Cu matrix to MWCNTs during the compressive strength test. The composites exhibited significant differences in orthogonal directions because the aligned MWCNTs had a larger contact area with the metal indenter in the parallel direction, which made for a more efficacious load transfer.

The elongation of the composite samples decreased. The elongation from high to low was the B-CNTs/Cu composite, Cu@CNTs/Cu composite, Ni@CNTs/Cu composite, and Ni&Cu@CNTs/Cu composite. The reduced elongation was attributed to the deformation during extrusion and drawing and the blocking effect of CNTs embedded in the matrix on dislocation slip [23]. The B-MWCNTs/Cu composite had the best ductility, which may be due to the agglomeration and entanglement of MWCNTs in the composite, which did not give full play to its role and did not strengthen the composite or reduce its ductility much. The better ductility of the Cu@CNTs/Cu composite was due to its reduced grain size (2.37 ± 1.03 μm), which was smaller than the 3.32 ± 1.1 μm of pure Cu. Its coherent small-scale twinning boundary provided significant strengthening and improved ductility [71]. In addition, low-angle GBs also improved the ductility of metallic materials due to the easier movement of dislocations through adjacent grains with slightly misoriented lattices [9].

The mechanical properties of the composite samples were orthogonal anisotropic. The strength parallel to the drawing direction was higher than that perpendicular to the drawing direction. Here, coated MWCNTs were straight, long, uniform dispersion, and unidirectionally aligned along a uniform direction in the Cu composite as shown in Figure 3, which could generate and store dislocations without causing cracks in GBs, facilitating the full utilization of the longitudinal load-bearing capacity of MWCNTs [23]. The curvature of MWCNTs in Cu matrix had a strong impact on the properties of composite. Savvas [72] calculated the response statistics of the apparent properties of polymer matrix composites by a combination of computational homogenization and Monte Carlo simulations, confirming that composites with oriented CNTs had greater axial stiffness than those with random CNTs and that the bending degree of CNTs had a more important effect on the stiffness of the composites.

## 6. Effect of the Interface on Conductivity

Good interfacial bonding enables the good transfer of electrons between CNTs and the matrix. The electron–phonon coupling is promoted, and the scattering of electrons and phonons at the interface is reduced, thereby improving the conduction properties of the overall composites [73]. However, the energy scattering at the interface becomes critical to the conductivity of composites. Well-dispersed MWCNTs increase the interfacial resistance at the scattering center and the interfacial dispersion of free electrons. The relationship between conductivity and interfacial scattering resistivity is large. According to the Fuchs size effect theory [74], the interfacial scattering resistivity *ρ*_int_ can be determined by the following equation:

(8) $$\rho_{\mathrm{int}}=\frac{3}{4}\frac{{\left(3n^{2}\right)}^{\frac{1}{3}h}}{e^{2}}\left(1-p\right)\frac{V_{\mathrm{CNTs}}}{\left(1-V_{\mathrm{CNTs}}\right)d}n^{\frac{-2}{3}}$$

where *h* is the reduced Planck constant; *e* is the charge of an electron; *p* is the probability of elastic scattering at the interface; *n* is the concentration of electron in Cu, and *d* is the diameter of CNTs.

The elastic scattering of electrons can be lessened by optimizing the interfacial structure, and the resistivity of interfacial scattering increases with wettability (*p*). To mitigate the effect of interfacial scattering, improving interface wettability is considered to be an efficacious method. Here, the interface between MWCNTs and the Cu matrix was bonded closely without gaps through the process of electroless plating, sintering, extrusion, and drawing, which reduced the resistivity of interfacial scattering and thus improved the overall conductivity of the MWCNTs/Cu composites.

The conductivity of the composites decreased with the addition of MWCNTs. The conductivity from high to low was the B-MWCNTs/Cu composite, Cu@CNTs/Cu composite, Ni&Cu@CNTs/Cu composite, and Ni@CNTs/Cu composite. This is opposite to the strength order of the composites. The reasons for this include (1) the larger size of MWCNTs caused disturbance of Cu lattice periods, causing the scattering of electrons in the local elastic strain regions, thereby reducing the conductivity. (2) High-density dislocations were generated in and around the interfacial regions after hot extrusion and cold drawing, and the heterogeneous interfaces increased, which led to the decrease in conductivity. As shown in Figure 6a,c, there were a large number of dislocation-intensive regions at both ends of MWCNTs, which seriously hindered the transmission of electrons and sounds. This might be the reason for the poor axial conductivity of the Ni@CNTs/Cu composite. (3) Although the oxide, carbide, and Ni coating at the interface of the composites could effectively transfer the load, their ability to transfer electrons and phonons was very limited, but acted as an interface electrical/thermal resistance, reducing conductivity. The decrease in the conductivity of the B-MWCNTs/Cu composite was the smallest because MWCNTs and the Cu matrix were closely bonded without gaps and other defects. The tight interface reduced the energy loss of electron–phonon conversion. In addition, the low-density dislocation near the interface reduced the Cu lattice disorder of amorphous regions, so the interface had a little negative impact on the conductivity of the B-MWCNTs/Cu composite.

The composite samples exhibited orthogonal anisotropy in conductivity, and the conductivity parallel to the tensile direction was obviously higher. This was attributed to the good axial conductivity of MWCNTs and the fact that MWCNTs remained straight, long, monodispersed, and had unidirectional arrangement in a uniform direction in the matrix. The bending degree of MWCNTs in the matrix had a strong influence on the properties of composites, and bending and torsion increased the scattering during the conduction of heat flow and current. The contact modes between CNTs and matrix could be divided into end contact and side contact [75]. The contact resistance of the latter is larger than that of the former in MWCNTs/Cu composites [76]. Due to the oriented arrangement of CNTs in the matrix, the electron transport along the interface perpendicular to the drawing direction was primarily impacted by the side contact mode, resulting in less conductivity [77,78] At the same time, the average free range of MWCNTs is long when conducting current because of their large aspect ratio [79]. Although the acid treatment produced defects on the outermost tubes of MWCNTs, intact inner tubes prevent the influence of electron/phonon transmission in the axial direction due to their multilayer nested tubular structure. When the contact at the interface was close, the electrons could be transmitted through the middle of MWCNTs. The electrons traveled a longer distance through MWCNTs aligned along the drawing direction compared to MWCNTs aligned perpendicular to the drawing direction, reducing the scattering effect of the interface on electrons and thus improving the conductivity of the composites [80].

## 7. Conclusions

We investigated the effect of Ni, Cu, and Ni&Cu electroless coatings on the interface between CNTs and the matrix and revealed the mechanism of interface composition and microstructure on the mechanical properties, conductivity, and ductility of MWCNTs/Cu composites.

1. The wettability of MWCNTs with respect to the Cu matrix as limited, resulting in weak mechanical bonding between CNTs and the Cu matrix in the B-MWCNTs/Cu composite. Therefore, its strengthening effect on the matrix was much lower than that of coated MWCNTs. However, the conduction performance of the B-MWCNTs/Cu composite had the minimum reduction, which may be ascribed to the reduction of the energy loss of electron–phonon conversion by the tightly bonded interface. In addition, the low-density dislocation near the interface also reduced the lattice disorder of Cu in amorphous regions.
2. There were interfacial products such as Cu_2_O and Ni_3_C, solid solutions of Cu and Ni, transitional amorphous areas, and high-density dislocations at interfacial regions in-plated CNT-reinforced Cu composites, forming tight interface bonding, which effectively strengthened the composites. However, these factors also caused the scattering of electrons and phonons, increasing the electrical/thermal resistance of the interface and leading to a decrease in the conductivity of composites. The ductility of the composites decreased, which was attributed to the blocking effect of MWCNTs on the dislocation slip.
3. The Ni plating was the most dense, continuous, and complete. The strengthening effect of Ni@CNT with respect to the Cu matrix is the greatest, but its elongation and conductivity decreased greatly. The Cu@CNTs/Cu composite achieved the balance of mechanical properties, ductility, and conductivity/thermal conductivity. Its ultimate tensile strength was 373 MPa, the elongation was 12.1%, and the axial conductivity and thermal conductivity were 79.9 IACS% and 376 W/mK, respectively.
4. MWCNTs in the prepared Cu composites remained straight, long, monodispersed, and unidirectionally arranged, which enabled the excellent axial conductivity and mechanical properties of MWCNTs to be exerted, exhibiting orthotropic anisotropy.
5. Even if the outermost tubes were damaged by acid treatment, MWCNTs with multilayer nested tube structures could still transmit electrons/phonons axially through inner tubes, which prevented the influence of outer tube damage on the conductivity.

## Figures and Tables

**Table 1:** Formula and conditions of electroless Ni plating.

**Formula (g/L)**	NiSO_4_ ·6H_2_O	40
	C_6_H_5_Na_3_O_7_	60
	NaH_2_PO_2_	20
	NH_4_Cl	35
	Activated MWCNTs	0.6
**Condition**	Temperature (°C)	20
	pH	8.0

**Table 2:** Formula and conditions of electroless Cu plating.

**Formula (g/L)**	CuSO_4_·5H_2_O	7.5
	C_2_H_2_O_3_ (50%)	27.5
	EDTANa_2_·2H_2_O	89.4
	2,2′-Dipyridyl	0.01
	Activated MWCNTs	0.6
**Condition**	Temperature (°C)	20
	pH	11.5

**Table 3:** Formula and conditions of electroless Ni&Cu plating.

**Formula (g/L)**	CuSO_4_·5H_2_O	7.5
	NiSO_4_·6H_2_O	0.75
	NaH_2_PO_2_	0.375
	C_2_H_2_O_3_	27.5
	EDTANa_2_·2H_2_O	89.4
	2,2′-Dipyridyl	0.01
	Activated MWCNTs	0.6
**Condition**	Temperature (°C)	20
	pH	11.5

**Table 4:** Test data of tensile, compressive, and conductive properties of samples.

		Cu	B-CNTs/Cu Composite	Cu@CNTs/Cu Composite	Ni&Cu@CNTs/Cu Composite	Ni @CNTs/Cu Composite
UTS (MPa)		259	264	332	373	391
YS (MPa)		231	250	309	316	358
Elongation (%)		18.5	16.2	12.1	9.50	7.50
UCS (MPa)	*F*_∥_	356	397	422	451	473
	*F*_⊥_	352	385	396	413	430
Thermal Conductivity (W/mK)	*C*_∥_	386	379	376	358	354
	*C*_⊥_	384	340	328	312	301
Electrical Conductivity (IACS%)	*C*_∥_	90.1	81.4	79.9	76.1	75.2
	*C*_⊥_	89.9	72.1	70.8	65.5	63.7

**Table 5:** Results of EBSD analysis.

Materials	Average Grain Sizes (μm)	Low-Angle GBs (%)
Cu	3.21 (±1.1)	41
B-CNTs/Cu composite	3.01 (±0.95)	49
Cu@CNTs/Cu composite	2.37 (±1.01)	54
Cu&Ni@CNTs/Cu composite	2.21 (±0.97)	57
Ni@CNTs/Cu composite	2.09 (±0.9)	61

## Data Availability

The data presented in this study are available on request from the corresponding author.
